# Attenuated β2 Integrin Expression in Neutrophils Alters Dendritic Cell Stimulation and T Cell Activation in Old Mice

**DOI:** 10.3390/cells15201852

**Published:** 2026-10-12

**Authors:** Christoph Hieber, Stephan Grabbe, Matthias Bros

**Affiliations:** 1 Department of Dermatology, University Medical Center of the Johannes Gutenberg—University Mainz, Langenbeckstraße 1, 55131 Mainz, Germany; chieber@uni-mainz.de (C.H.); stephan.grabbe@unimedizin-mainz.de (S.G.); 2 Institute of Molecular Biology (IMB), Ackermannweg 4, 55128 Mainz, Germany

**Keywords:** β2 integrins, neutrophil, PMN, dendritic cell, T cell, aging

## Abstract

Polymorphonuclear neutrophils (PMNs) are critical for defense against bacterial and fungal pathogens, relying on the β2 integrins LFA-1 and MAC-1 to mediate adhesion, phagocytosis, ROS production, and NETosis. β2 integrins are heterodimeric surface receptors that are composed of a variable α (LFA-1: CD11a, MAC-1: CD11b) and CD18 as the constant β subunit. We have previously shown that in mice, partial PMN-specific CD18 knockdown worsens aspergillosis by impairing PMN function. Given that aging also alters PMN behavior, this study aimed to elucidate whether reduced β2 integrins in PMNs of aged mice affect their interactions with other immune cells. Comparing young (8–12 weeks) and aged (>52 weeks) mice, we observed that PMN-specific CD18 reduction remained stable across age groups, with CD18 expression reduced to approximately 60–70% of WT levels. PMNs from aged CD18^Ly6G^ mice showed reduced phagocytosis of *A. fumigatus* conidia and increased CD86 expression following stimulation. Regarding extrinsic effects, aging was associated with increased MHC class II expression on splenic myeloid cells and an activated T-cell phenotype. Polyclonal stimulation resulted in increased TNF-α production in cultures of splenocytes derived from aged CD18^Ly6G^ mice, and CD4^+^ T cells were characterized by reduced induction of Th1- and Th2-associated transcription factors. DCs from aged CD18^Ly6G^ mice showed altered activation and cytokine secretion and induced less antigen-specific CD4^+^ T-cell proliferation. In addition, CD18-knockdown PMNs of aged mice stimulated dendritic cells (DCs) less effectively than WT PMNs. Thus, partial β2 integrin reduction in PMNs impairs selected PMN effector functions and is associated with altered DC activation and CD4^+^ T-cell responses during aging.

## 1. Introduction

Polymorphonuclear neutrophils (PMNs) constitute 50–70% of circulating white blood cells in humans, serving as a crucial first line of defense against invading pathogens [1]. PMNs arise from common myeloid progenitors and have a relatively short lifespan at steady state, which is increased under inflammatory conditions [1,2]. While PMN numbers seem relatively stable in both mice and humans during aging, a number of functional alterations have been described [3]. Several studies reported reduced chemotaxis of PMNs in older humans and mice upon infection [4,5,6]. In this regard, PMN trafficking from the circulation to sites of inflammation is a tightly regulated process crucial for effective pathogen clearance [7]. While some studies report age-dependently reduced migratory capacity and speed of PMNs, others have observed enhanced trafficking to organs such as the lungs and liver in old mice following infection [5,6,8,9,10,11]. Leukocyte rolling and extravasation into inflamed tissue requires binding of β2 integrins to intercellular adhesion molecule (ICAM)1 on endothelial cells [12]. While ICAM is rather widely expressed, β2 integrins are exclusively apparent on leukocytes. They consist of a variable α subunit (CD11a-CD11d) that pairs with the constant β2 subunit CD18, thereby forming heterodimers [13]. Murine and human PMNs express LFA-1 (Lymphocyte function-associated antigen 1, CD11a/CD18) and MAC-1 (Macrophage-1 antigen, CD11b/CD18) that are important for extravasation [14]. While LFA-1 mediates initial adhesion and arrest, MAC-1 stabilizes binding to the endothelium and confers crawling. Consequently, PMN extravasation is significantly impaired in CD18-deficient mice [15]. In contrast, during pneumonia, PMN emigration was even increased in these mice as compared to their wild-type counterparts [16]. Elevated PMN infiltration in the lungs was also observed by us in CD18- and CD11b-deficient mice in invasive pulmonary aspergillosis [17,18].

Furthermore, MAC-1 and CD11c/CD18 are essential for phagocytosis of iC3b opsonized pathogens [19]. In line, PMNs from patients suffering from hereditary loss of CD18 showed reduced phagocytic activity, which can be mimicked in vitro using CD11b and CD18 blocking antibodies [20]. Another key mechanism of host defense by PMNs is the formation of neutrophil extracellular traps (NETs), web-like structures consisting of fibers, DNA, and granule proteins [21]. The ability to form these NETs in response to pathogen challenge, termed NETosis, has been shown to be impaired in aged mice [22]. In contrast, in humans, an increase in NET formation in response to lipopolysaccharide (LPS) has been observed for PMNs from older donors compared to those derived from younger individuals. However, the antibacterial activity was lower in PMNs from the elderly donors [23]. Behnen and coworkers showed that MAC-1 in combination with FCγRIIIb is important for NET formation [24]. In line with this, MAC-1 and downstream outside-in signaling were shown to be indispensable for reactive oxygen species (ROS)-dependent NETosis following *A. fumigatus* infection [25]. ROS also constitute a direct, important antimicrobial effector mechanism of PMNs [26]. Ogawa and coworkers observed elevated spontaneous and stimulus-triggered ROS production in PMNs from elderly donors compared to younger ones, which may contribute to oxidative stress and drive inflammaging [27]. Furthermore, Butcher et al. reported a diminished phagocytic potential of PMNs derived from elderly donors [28]. Moreover, older individuals displayed higher frequencies of the immunosuppressive CD16^bright^/CD62L^dim^ PMN subpopulation compared to young donors, associated with reduced phagocytic activity and ROS production [29]. We also showed impaired ROS generation and NET formation in PMNs with reduced CD18 expression after stimulation with *A. fumigatus* [17].

This study aimed to investigate whether age-associated alterations in PMN function are exacerbated by reduced β2 integrin expression and how PMN-specific CD18 deficiency influences PMN-dendritic cell interactions and subsequent T-cell responses.

## 2. Materials and Methods

### 2.1. Mice

Mice were bred and maintained under specific pathogen-free conditions at the Central Animal Facility of the Johannes Gutenberg University Mainz. CD18^fl/fl^ mice [30] were crossed with Ly6G-Cre [31] to generate CD18^Ly6G^ conditional knockout (cKO) mice as described [17]. Mice were sacrificed according to §4 of the German Animal Welfare Act (TierSchG). Analyses compared CD18^fl/fl^ as wild-type (WT) controls to cKO mice in young adults (8–12 weeks) and aged mice (52–66 weeks). Male and female mice were included in equal numbers within each age- and genotype-defined group but were not distinguished in the subsequent analyses.

### 2.2. Cell Isolation

Spleens were homogenized through 40 µm cell strainers, washed in 25 mL wash medium (Minimal Essential Medium + 5% FBS + 1% penicillin/streptomycin), centrifuged (300× *g*, 5 min), and treated with hypotonic lysis buffer (1 min, RT) to remove erythrocytes. Cells were washed, centrifuged, and resuspended in test medium (Iscove’s Modified Dulbecco’s Medium + 5% FBS + 1% penicillin/streptomycin 1% β-mercaptoethanol, 1% L-glutamine). Media and supplements were purchased from Sigma-Aldrich (St. Louis, MO, USA). For DC isolation, spleens were cut into small pieces and digested with 2 mg/mL Collagenase D in Hank’s balanced salt solution (Sigma-Aldrich) for 30 min at 37 °C before passing through the cell strainers. To isolate bone marrow, tibiae, femurs, and ilia were disinfected in 70% ethanol (1 min), washed in PBS (Sigma-Aldrich), and flushed with 23G needles into wash medium (minimal essential medium, supplemented with 5% FBS + 1% penicillin/streptomycin). Cells were filtered through 70 µm strainers and processed as above.

### 2.3. Cell Cultures

PMNs were enriched from bone marrow using the Myeloid-Derived Suppressor Cell Isolation Kit (Miltenyi Biotec, Bergisch-Gladbach, Germany) and were cultured in test medium. The purity of isolated cells is shown in Supplementary Figure S1. To obtain bone marrow-derived DC (BMDC), bone marrow cells (2 × 10^6^ cells/mL) were seeded with 1 mL test medium, supplemented with 250 ng/ mL FLT3L Biolgend (San Diego, CA, USA, and cultured for 7 days at 37 °C, 7.5% CO_2_. Differentiated DCs were subjected to experiments on days 7–8 of culture. Spleens were digested with 2 mg/mL Collagenase D (30 min, 37 °C), filtered, and primary DCs were enriched using theBiolegend) MojoSort Mouse Pan Dendritic Cell Isolation Kitas recommended. The purity of isolated cells is shown in Supplementary Figure S1. For the co-culture of PMNs with FLT3L-derived BMDCs, 10^6^ BMDC from one female WT mouse were each cultured with 3x10^6^ PMNs of the different age and genotype groups. All gating strategies are depicted in the supplementary figures.

### 2.4. Functional Assays

To assess phagocytic uptake, PMNs were seeded into 96-well plates (10^5^/100 µL), incubated with green fluorescent protein (GFP)-expressing *Aspergillus fumigatus* conidia (AfS148) for 1 h at 37 °C, washed, and analyzed by flow cytometry for phagocytic uptake.

### 2.5. T Cell Stimulation

PMNs and DCs were pulsed with OVA 257–264 (SIINFEKL) or OVA 323–339 peptide (both InvivoGen, San Diego, CA, USA) (each 100 ng/ml) for 2 h, washed, and co-cultured with OVA antigen-specific splenic CD8^+^ (strain OT-I) or CD4^+^ (OT-II) T cells obtained by immunomagnetic sorting (CD8a (Ly-2) MicroBeads, mouse, CD4 (L3T4) MicroBeads, mouse, Miltenyi) for 2–4 days. T cell proliferation was assessed using two different methods: CellTrace Violet (CTV) (ThermoFisher Scientific, Waltham, MA, USA) dilution using flow cytometry and ^3^H-thymidine incorporation. For the CTV-based analysis, 10^6^ T cells were labeled with 1 µL CTV in 1 mL PBS for 20 min at room temperature prior to co-culture, followed by washing with 50 mL of medium. CTV-labeled T cells were then used in co-culture experiments as described above. For presentation, CTV mean fluorescence intensity (MFI) was expressed as its reciprocal (1/MFI), so that higher values corresponded to greater dye dilution and thus more T cell divisions. The resulting values were normalized to the mean 1/MFI of the young WT group. For the radioactive proliferation assay, unlabeled T cells were co-cultured with APC and ^3^H-thymidine (Revvity, Waltham, MA, USA) (0.5 µCi/well) was added for the final overnight incubation. After harvesting and washing, incorporated radioactivity was quantified using a microplate β counter.

### 2.6. Flow Cytometry and Cytokine Analysis

Antibodies were purchased from BioLegend, ThermoFisher, or BD Biosciences (San Jose, CA, USA). Samples were Fc receptor-blocked (clone 2.4G2; 10 min, 4 °C), incubated with surface antibodies (Table 1) for 30 min at 4 °C, and stained with fixable viability dye (ThermoFisher Scientific, Waltham, MA, USA) (20 min, dark). For intracellular targets, cells were fixed/permeabilized (Foxp3/Transcription Factor Staining Buffer Set; ThermoFIsher) and incubated with transcription factor-specific antibodies. Compensation was performed using single-stained controls. Data were acquired on an Attune NxT cytometer (ThermoFisher). Cytokines in cell culture supernatant were quantified using BD Biosciences (Franklin Lakes, NJ, USA) CBA Flex kits. Samples were incubated with capture beads and PE detection antibodies (1 h each, RT), washed, and analyzed via BD Biosciences FCAP Array software (v3.0).

### 2.7. Statistical Analysis

Data were analyzed using GraphPad Prism v9.5.1. Two-group comparisons used unpaired two-tailed t-tests; ≥3 groups used one-way/two-way ANOVA with Tukey’s or Šídák’s multiple comparisons tests as indicated. Significance: ns (*p* > 0.05), * *p* < 0.05, ** *p* < 0.01, *** *p* < 0.001, and **** *p* < 0.0001.

## 3. Results

### 3.1. Partial CD18 Loss Is Associated with Altered PMN Functions

We first confirmed that PMN-specific knockdown of CD18 resulted in a reduction in its surface expression in bone marrow-derived (Figure 1A) and splenic (Figure 1B) PMNs from both young and aged mice, with levels reduced to approximately 60–70% of those in WT controls. Other splenic leukocyte populations showed no genotype-dependent alteration in CD18 expression. Functional analysis revealed that aged CD18-deficient PMNs displayed impaired phagocytosis of *Aspergillus fumigatus* conidia (Figure 1C). Antigen processing was lower in PMNs with a CD18 knockdown at younger age, and PMNs obtained from old mice of either genotype presented with lower activity (Figure 1D). Upon stimulation, CD18^Ly6G^ cKO PMNs derived from aged mice exhibited a stronger increase in surface CD86 expression than WT controls (Figure 1E), and in line with this, CD62L expression trended lower. In accordance with an elevated state of activity, expression of MHC II was increased in PMNs obtained from old mice but showed no genotype-dependent differences, while MHC class I remained unchanged. Analysis of cytokine secretion from stimulated PMNs showed that age and CD18 knockdown correlated with higher levels of TNF-α but not CXCL10 (Figure 1F).

Stimulated PMNs can act as antigen-presenting cells that activate CD8^+^ T cells [32]. Due to the overall β2 integrin-dependent alterations in the immunophenotype of PMNs, we assessed their ability to stimulate antigen-specific CD8^+^ T cells. When pulsed with Ovalbumin (OVA)-derived SIINFEKL peptide and co-cultured with OVA antigen-specific OT-1 T cells, CD18-deficient PMNs tended to induce less T cell proliferation than WT PMNs (Figure 1G). However, the production of IFN-γ and TNF-α by T cells did not differ between the two groups (Figure 1G). 

Altogether, these findings suggest that moderate knockdown of β2 integrins on PMNs only moderately affects their immunophenotype in aging.

### 3.2. CD18-Deficient PMNs in Aged Mice Alter Splenic Immune Cell Activation and Skew T Cell Differentiation

To delineate potential extrinsic effects of β2 integrin-deficient PMNs, we examined the overall composition of splenic leukocyte populations but found no significant differences in the frequency of major immune subsets between young and aged mice, or between genotypes (Figure 2A). MHC II levels were generally elevated in PMNs and other myeloid populations derived from old mice, but no significant genotype-dependent differences were observed. Similar to bone marrow-derived PMNs, CD86 expression was significantly higher in unstimulated and LPS-stimulated CD18-knockdown PMNs derived from aged animals both unstimulated and after LPS stimulation. Macrophages from aged mice also showed a trend toward increased CD86. Interestingly, T cells presented with an age-associated more activated phenotype, characterized by higher CD44 and CD69 expression and concomitantly reduced CD62L levels (Figure 2B).

To evaluate cytokine responses, splenocytes were stimulated overnight with a TLR7/8 agonist and T cells were polyclonally stimulated, respectively, and production of TNF-α as a prototypic immediate-early innate cytokine was measured. R848-induced TNF-α production was lower in splenocytes from aged mice, regardless of their genotype. In contrast, the level of TNF-α generated by activated T cells was highest for cells derived from old CD18^Ly6G^ cKO mice (Figure 2C).

Analysis of T cell subset frequencies in the spleen revealed no genotype- or age-dependent differences regarding CD4^+^, CD8^+^, and γδ T cells. However, γδ T cells from aged CD18^Ly6G^ cKO mice showed a trend toward higher CD69 expression, indicative of a higher state of activity (Figure 2D). Next, we examined the capacity of polyclonal stimulated splenic CD4^+^ T cells to differentiate into distinct helper subsets. Under basal conditions, CD4^+^ T cells obtained from aged mice exhibited higher frequencies of Foxp3^+^ regulatory T cells. After one day of polyclonal stimulation, induction of Th1 and Th2 cells was lower in samples obtained from CD18^Ly6G^ cKO mice regardless of their age compared to their WT counterparts. These findings suggest that although PMNs themselves may not exert considerable CD4^+^ T cell stimulatory activity under steady-state conditions, PMN-restricted β2 integrin deficiency has an impact on the responsiveness of CD4^+^ T cells towards activation.

### 3.3. PMN-Specific CD18 Knockdown Alters Splenic DC Phenotype and Is Associated with Reduced CD4^+^ T-Cell Priming in Aged Mice

It has previously been demonstrated that PMNs interact with DCs, regulating their T cell stimulatory capacity [33]. In turn, T cell homeostasis is largely controlled by dendritic cells (DCs) that present antigens in the periphery, inducing tolerance in the absence of pathogen-derived and endogenous danger signals but evoke strong T cell responses when activated [34]. In CD18^Ly6G^ cKO mice, frequencies of conventional (c)DC1, cDC2, and plasmacytoid (p)DCs were comparable across all experimental groups (Figure 3A). To evaluate potential functional alterations, splenic DCs were pulsed with OVA-derived peptides and co-cultured with OVA-specific CD4^+^ (OT-2) and CD8^+^ (OT-1) T cells, respectively. We observed that DCs obtained from aged CD18^Ly6G^ cKO mice showed a trend towards lower induction of CD4^+^ T-cell proliferation than DCs derived from old WT animals, while CD8^+^ T-cell proliferation remained unchanged across all groups (Figure 3B).

We observed no genotype-dependent differences regarding MHC class II and CD86 expression by DCs. In contrast, PD-L1 expression was elevated on LPS-stimulated cDC2 from aged CD18Ly6G cKO mice (Figure 3C). This difference was significant compared to the young groups and showed an upward trend compared to the older WT group. In addition, CD18^Ly6G^ cKO DCs isolated from spleens of old mice secreted less TNF-α, IL-6, IFN-β, and CCL5 than the other groups, whereas no differences were detected in DCs obtained from young mice of either genotype (Figure 3D). These observations suggest that a PMN-specific knockdown of β2 integrins has an impact on the immunophenotype of DCs, specifically in aged mice.

### 3.4. CD18-Deficient PMNs Show Impaired Capacity to Stimulate DCs

To determine whether murine PMNs may directly affect the DC immunophenotype in an age-dependent manner via ß2 integrins, FLT3L-derived BMDCs were co-cultured overnight with primary PMNs isolated from young and aged WT or CD18^Ly6G^ cKO mice, respectively. DC viability was comparable across all conditions (Figure 4A). In general, co-culture with PMNs enhanced the activation state of cDC1s and cDC2s, as reflected by increased CD86 expression (Figure 4B). However, co-culture of DCs with PMNs derived from aged CD18Ly6G cKO mice resulted in significantly lower CD86 upregulation in cDC1s compared with co-cultures containing PMNs from aged WT or young CD18Ly6G cKO mice. A similar trend was observed for cDC2s and for MHC class II expression in cDC1s. In contrast, activation of pDCs was largely unaffected. Analysis of cytokine secretion in co-culture supernatants revealed that TNF-α and IL-6 levels were lower in co-cultures containing PMNs from aged CD18^Ly6G^ cKO mice compared with the other groups, albeit below statistical significance (Figure 4C).

In summary, CD18 knockdown in aged PMNs results in increased PMN activation and reduced phagocytosis but does not impair PMN-mediated CD8^+^ T cell stimulation. Furthermore, PMNs may activate DCs in a β2 integrin-dependent manner, resulting in disturbed T cell homeostasis as reflected by altered responsiveness of T cells to stimulation.

## 4. Discussion

Aging is associated with major changes in PMN function that may compromise host defense. These changes include impaired chemotaxis, reduced phagocytic capacity, and altered generation of reactive oxygen species production collectively contributing to the declining antimicrobial immunity observed in elderly individuals [2,3,4]. Since β2 integrins regulate key PMN functions, including adhesion, migration, and phagocytosis, altered β2 integrin expression may be particularly relevant during aging [19,35]. As complete CD18 deficiency affects many leukocyte populations [15], CD18^Ly6G^ cKO mice provide a useful tool to investigate how reduced CD18 expression specifically in PMNs influences immune function during aging. To study age-dependent consequences of CD18 reduction in PMNs, we characterized young and aged CD18^Ly6G^ cKO mice alongside their WT littermates. In the former, CD18 expression was attenuated to approximately 65% of WT levels specifically in PMNs, and this reduction remained stable across age groups (Figure 1A), as previously reported by us for young animals [17]. Thus, this model allows the effects of age to be studied in the context of a stable, partial reduction in PMN β2 integrin expression.

Partial CD18 reduction affected selected PMN functions, most prominently in aged mice. CD18 reduction in PMNs impaired their phagocytic and antigen processing activity (Figure 1C,D), supporting an inverse relationship between MAC-1 expression and phagocytic capacity, especially in PMNs derived from aged mice. CD18-deficient PMNs obtained from aged CD18^Ly6G^ cKO mice exhibited significantly increased CD86 and a trend towards reduced CD62L expression after stimulation compared to WT (Figure 1E), suggesting that CD86 expression may be negatively regulated by β2 integrin activity. Whether β2 integrin signaling actively inhibits CD86 expression or whether increased CD86 expression represents a compensatory mechanism in aged β2 integrin-deficient PMNs needs to be further investigated. However, CD18 knockdown did not significantly impair PMN-mediated CD8^+^ T cell proliferation or cytokine production (Figure 1G). We have previously shown that β2 integrins on myeloid DCs [36,37] and macrophages [36] limit T cell activation. Hence, attenuated expression of β2 integrins on myeloid PMNs may not confer detrimental direct effects regarding T cell activation.

Surprisingly, T cells from aged CD18^Ly6G^ cKO mice displayed increased CD44 and CD69 expression and reduced CD62L expression, consistent with an accumulation of activated or antigen-experienced cells [2,38] (Figure 2B). Thus, the absence of a detectable effect in the direct PMN–CD8+ T cell assay did not rule out broader changes in the T cell compartment of aged mice. R848-induced TNF-α production was lower in splenocytes from aged mice independent of their genotype, indicating an age-associated change in the responsiveness of the total splenocyte population (Figure 2C). In contrast, polyclonal T cell stimulation resulted in significantly elevated TNF-α levels in cultures of cells derived from aged CD18^Ly6G^ mice. It remains unknown whether these observations reflect the whole T cell compartment or are driven by a specific subset. Furthermore, somewhat surprisingly, we observed that polyclonally stimulated CD4^+^ T cells from CD18^Ly6G^ cKO mice showed decreased potential toward Th1 and Th2 differentiation (Figure 2E).

The impaired CD4^+^ T-cell response may result from altered PMN–DC crosstalk. DCs isolated from aged CD18^Ly6G^ cKO mice induced less OT-II CD4^+^ T-cell proliferation than DCs from the corresponding WT mice, whereas OT-I CD8^+^ T-cell proliferation remained unaffected (Figure 3B). In parallel, LPS-stimulated cDC2s from aged CD18-knockdown mice showed increased PD-L1 expression and reduced secretion of TNF-α, IL-6, IFN-β, and CCL5 (Figure 3C,D). As DC subset frequencies were unchanged (Figure 3A), these observations indicate an altered functional state rather than a numerical defect of splenic DCs. Our PMN–DC co-culture experiments employing in vitro-differentiated DCs previously shown to present with higher fitness than isolated primary DCs supported this hypothesis. PMNs generally enhanced CD86 expression on FLT3L-derived BMDC, whereas PMNs from aged CD18^Ly6G^ cKO mice induced significantly less DC activation than PMNs from aged WT mice (Figure 4B). Similar trends were observed for MHC class II expression and cytokine release, while DC viability and pDC activation were largely unaffected (Figure 4A-C). Further experiments are necessary to confirm this interaction. Together, these findings suggest that partial CD18 reduction can diminish the ability of aged PMNs to promote DC activation.

It has been shown that MAC-1 on PMNs interacts with DC-SIGN, promoting DC activation [33]. It is conceivable that impaired β2 integrin expression by PMNs may affect PMN-DC crosstalk, leading to attenuated DC maturation and inadequate CD4^+^ T cell activation. Future studies should determine the mechanism by which reduced PMN CD18 affects DC activation, in particular whether the effect depends on direct PMN–DC contact or is mediated by soluble factors.

In conclusion, our findings suggest that β2 integrins are necessary to maintain PMN function during aging since PMN-specific CD18 reduction affected phagocytosis and activation marker expression while largely preserving CD8^+^ T cell priming capacity. Nevertheless, PMN-restricted β2 integrin knockdown was associated with altered T cell activation in aged mice, including changes in helper T cell differentiation presumably due to impaired DC activation by PMNs. Together, these findings highlight a dose-dependent and function-specific role of β2 integrins in coordinating neutrophil effector functions and shaping (adaptive) immune responses during aging.

## Figures and Tables

**Figure 1:**
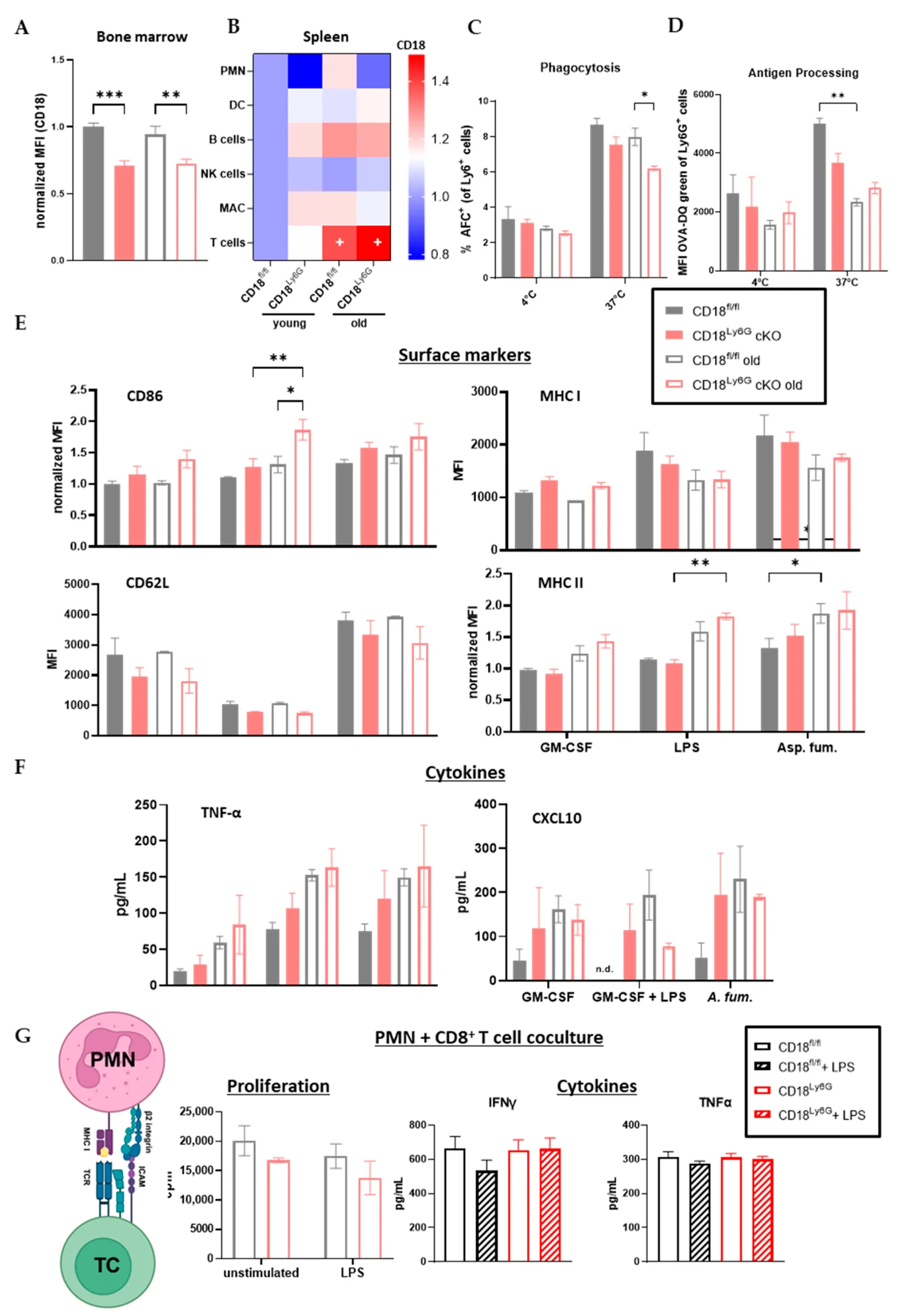
Partial CD18 loss in PMNs impairs phagocytosis, alters activation, and reduces T cell priming in aged mice. CD18 surface expression was quantified by flow cytometry on (AMACS-purified bone-marrow PMNs (mean ± SEM, n = 7–8) and (**B**) splenocytes (n = 3) from (**A**,**B**) young (8–12 weeks) and aged (>52 weeks) CD18^fl/fl^ and CD18^Ly6G^ cKO mice. Mean fluorescence intensity (MFI) values were normalized to the mean of the CD18^fl/fl^ young group. Statistical significance between old and young are depicted. (**C**) Phagocytosis of GFP-expressing *A. fumigatus* conidia and (**D**) antigen processing capacity, assessed using DQ-OVA, by PMNs immunomagnetically sorted from bone marrow were measured by flow cytometry after 1 h incubation at 4 °C and 37 °C to differentiate between binding and uptake and to assess background. Data denote the mean ± SEM (n = 3). (**E**) Bone marrow-derived PMNs were cultured overnight with 100 ng/mL GM-CSF alone, or in combination with 1 µg/mL LPS or with *A. fumigatus* conidia at a 1:1 ratio. Surface expression of CD86, CD62L, MHC II, and MHC I was analyzed by flow cytometry (mean ± SEM, n = 3–5). (**F**) Supernatants from the PMN stimulation cultures were analyzed for TNF-α and CXCL10 concentrations by CBA. (mean ± SEM, n = 3) (**G**) Bone marrow-derived PMNs obtained from aged animals were left untreated or stimulated with 1 µg/mL LPS overnight. The next day, they were pulsed with 100 ng/mL SIINFEKL peptide and co-cultured with OT-I CD8^+^ T cells (1:2 ratio) for 48 h. Left panel: Schematic overview of PMN/T cell co-culture, created with BioRender.com. Middle panel: T cell proliferation was measured by ^3^H-thymidine incorporation (cpm). Right panel: IFN-γ and TNF-α in co-culture supernatants were quantified by CBA. (mean ± SEM, n = 3). (**A**–**G**) All experiments were performed independently with biological replicates. Statistical analysis was performed using one-way ANOVA with Tukey’s post hoc test; +/* *p* < 0.05, ** *p* < 0.01, and *** *p* < 0.001, n.d. = not determined (values below detection threshold).

**Figure 2:**
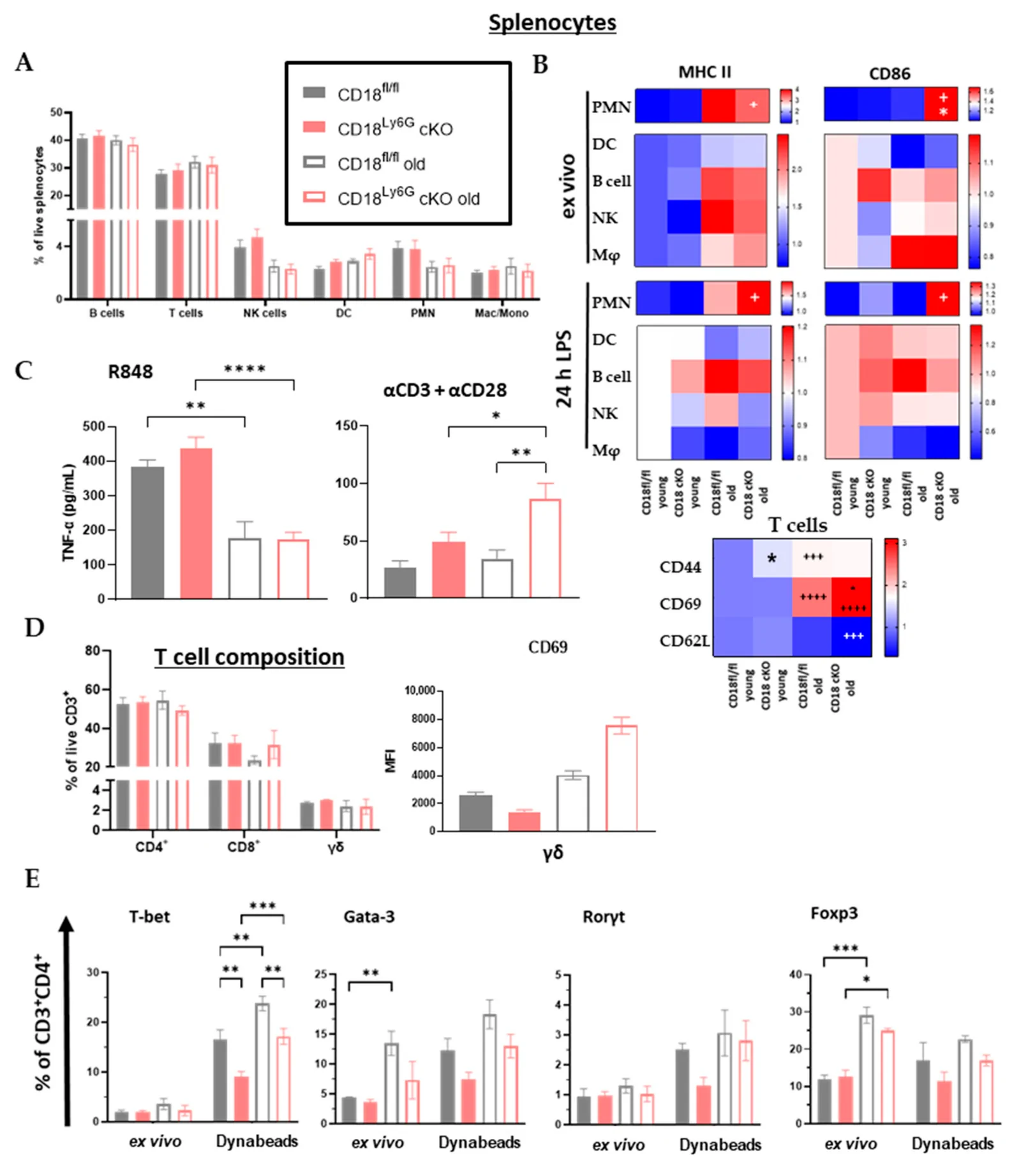
CD18-deficiency of PMNs in aged mice alters splenic immune cell activation and skews T cell differentiation. (**A**) Frequency of major leukocyte populations in the spleen was assessed by flow cytometry (mean ± SEM, n = 3 per group). (**B**) Activation state of splenic myeloid and lymphoid cell types. Upper panel: Heat maps show surface expression of MHC class II and CD86 on PMNs, macrophages, dendritic cells, NK cells, and B cells after isolation and after 24 h stimulation with 1 µg/mL LPS (n = 4–8). Lower panel: Surface expression of CD44, CD69, and CD62L on splenic T cells after isolation (n = 3). Statistical significance within age groups (CD18^fl/fl^ vs. CD18^Ly6G^) (*) and old vs. young (^+^) is depicted. (**C**) Splenocytes were stimulated overnight with 1 µg/mL R848 (left panel) or anti-CD3/CD28 polyclonal T cell activator beads (right panel), and TNF-α concentrations in supernatants were measured by CBA (mean ± SEM, n = 4–7). (**D**) Frequency of CD4^+^, CD8^+^, and γδ T cells in the spleen was analyzed by flow cytometry (n = 3). Right panel: CD69 expression on γδ T cells after isolation (mean ± SEM, n = 2). (**E**) Intracellular expression of transcription factors T-bet, Gata-3, RORγt, and Foxp3 was analyzed in freshly isolated splenic T cells and after 24 h of polyclonal stimulation (mean ± SEM, n = 4). (**A**–**E**) All experiments were performed independently with biological replicates. Statistical analysis was performed using one-way ANOVA with Tukey’s post hoc test; ^+/^* *p* < 0.05, ** *p* < 0.01, ^+++/^*** *p* < 0.001, ^++++/^**** *p* < 0.0001.

**Figure 3:**
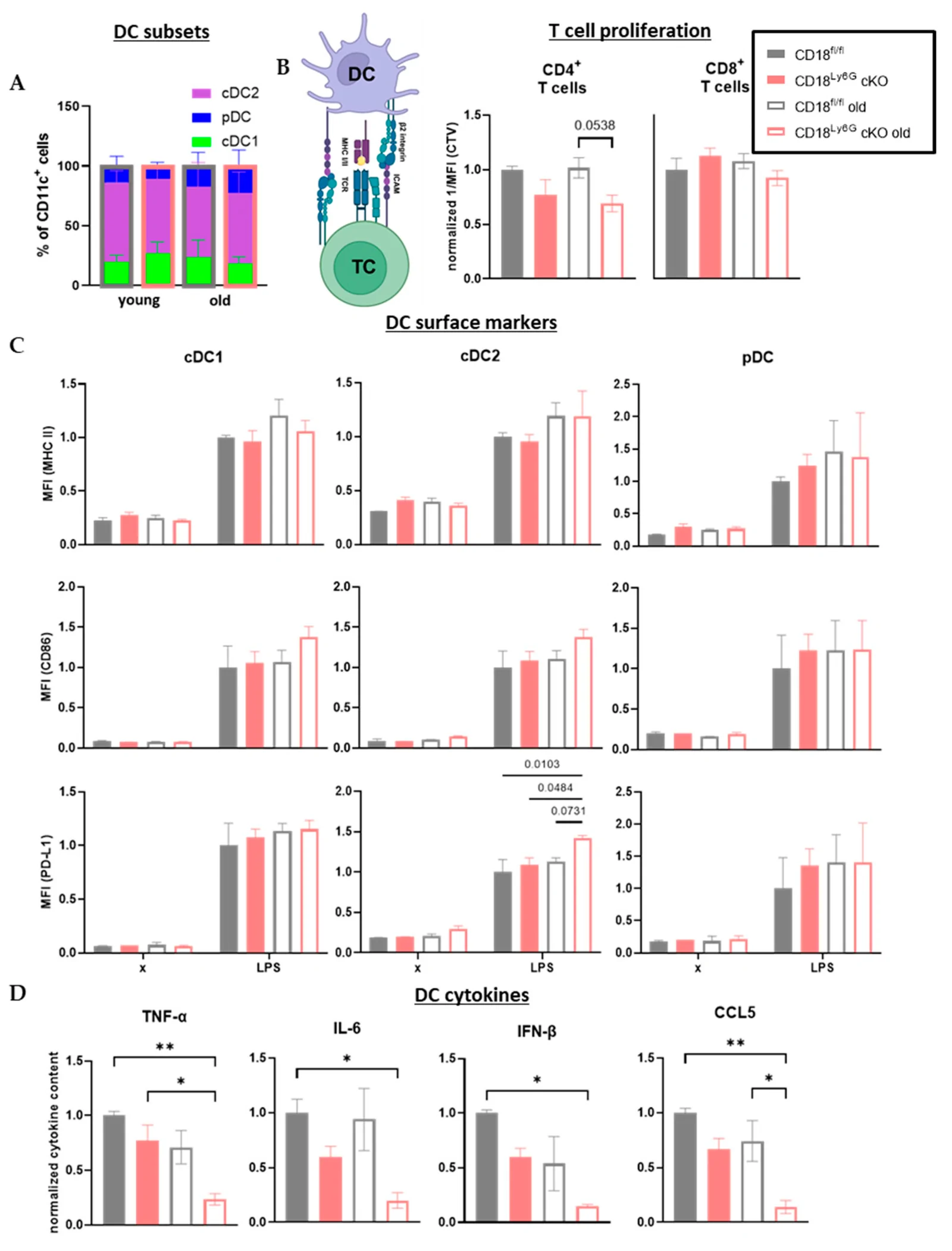
CD18-deficient DCs exhibit altered activation and T cell priming capacity. (**A**) Frequency of splenic DC subsets, including cDC1, cDC2, and pDC, was analyzed by flow cytometry (mean ± SEM, n = 3 per group). (**B**) Left panel: Schematic overview of DC/T cell co-culture, created with BioRender.com. Proliferation of CD4^+^ OT-II (middle panel) and CD8^+^ OT-I (right panel) T cells after 48–96 h co-culture with primary, OVA peptide-loaded splenic DCs was assessed by flow cytometry (mean ± SEM, n = 3–4). (**C**) Activation state of primary splenic cDC1, cDC2, and pDCs following overnight stimulation with 1 µg/mL LPS. Surface expression levels of MHC class II, CD86, and PD-L1 were determined by flow cytometry (mean ± SEM, n = 3–4). (**D**) Cytokine concentrations in supernatants of LPS-stimulated primary DC cultures from (**C**) were quantified by CBA (mean ± SEM, n = 3–4) and normalized to the young CD18^fl/fl^ group. (**A**–**D**) All experiments were performed independently with biological replicates. Statistical analysis was performed using one-way ANOVA with Tukey’s post hoc test; * *p* < 0.05 and ** *p* < 0.01.

**Figure 4:**
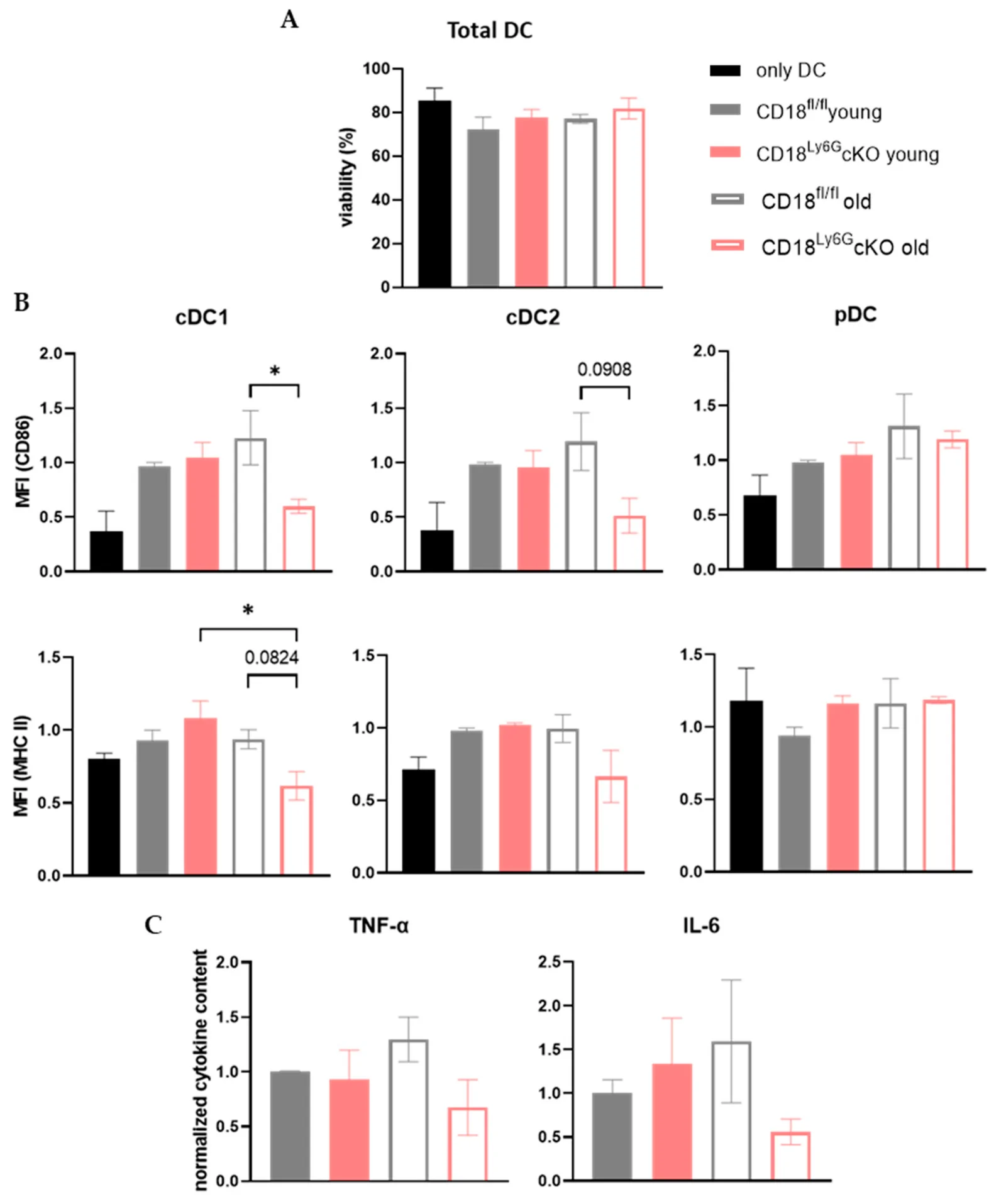
CD18-deficient PMNs alter DC activation. (**A**) Viability of DCs after overnight co-culture with PMNs (1:3 DC:PMNs) was assessed by flow cytometry (mean ± SEM, n = 3–4). (**B**) Activation state of BMDC subsets, including cDC1, cDC2, and pDCs after co-culture with PMNs. Surface expression of CD86 and MHC class II was analyzed by flow cytometry (mean ± SEM, n = 3–4). (**C**) Cytokine concentrations in co-culture supernatants were quantified by CBA and normalized to the young CD18^fl/fl^ group (mean ± SEM, n = 3–4). (**A**–**C**) All experiments were performed independently with biological replicates. Statistical analysis was performed using one-way ANOVA with Tukey’s post hoc test; * *p* < 0.05.

**Table 1:** Antibodies used for investigations.

Target	Dye
B220	Horizon V500
CD11b	SB600
CD11c	APC
CD172a	PE-Cy7
CD18	APC
CD19	SB702
CD3	PE-Cy5
CD4	FITC
CD62L	APC
CD86	FITC
Foxp3	FITC
Gata-3	APC
Ly6G	PE-eFl610
MHC I	PE
MHC II	eFl450
NK1.1	PE
PD-L1	PE-CF594
Rorγt	PE
T-bet	PerCP-Cy5.5
TCR γδ	APC
XCR1	AF647

## Data Availability

The original contributions presented in this study are included in the article. Further inquiries can be directed to the corresponding author.

## Supplementary Materials

The following supporting information can be downloaded at https://www.mdpi.com/article/10.3390/cells15201852/s1. Figure S1: Flow cytometric analysis of purity of PMN immunomagnetically sorted from bone marrow, Figure S2: Flow cytometric gating strategy of splenocytes, Figure S3: Flow cytometric gating strategy of CD4+ T cell subsets. Figure S4: Flow cytometric gating strategy of primary dendritic cell subsets. Figure S5: Flow cytometric gating strategy of CD4+ T cells after coculture with DCs.

## Abbreviations

The following abbreviations are used in this manuscript:

BMDC	Bone marrow-derived dendritic cell
c	Conventional
CBA	Cytometric bead array
CR	Complement receptor
DC	Dendritic cell
GFP	Green fluorescent protein
ICAM	Intercellular adhesion molecule
LFA-1	Lymphocyte function-associated antigen 1
LPS	Lipopolysaccharide
MAC-1	Macrophage-1 antigen
NET	Neutrophil extracellular trap
OVA	Ovalbumin
p	plasmacytoid
PMNs	Polymorphonuclear neutrophils
ROS	Reactive oxygen species
