# Supplementary material for: Attenuated β2 Integrin Expression in Neutrophils Alters Dendritic Cell Stimulation and T Cell Activation in Old Mice
Source: Cells. 2026 Oct 12;15(20):1852. doi: 10.3390/cells15201852 (PMC13702577; doi:10.3390/cells15201852)
Supplement: Supplementary file 1 [file cells-15-01852-s001.zip › cells-4572924-supplementary.pdf]

Supplementary Materials

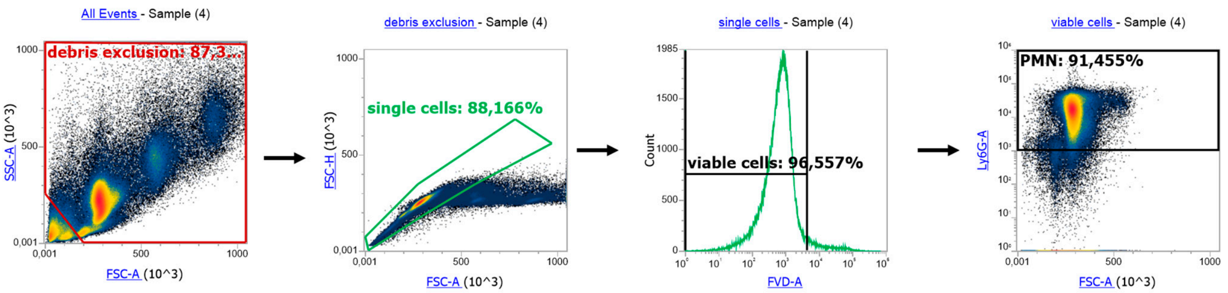

Figure S1. Flow cytometric analysis of purity of PMN immunomagnetically sorted from bone marrow. Representative density plots and histograms are shown. After debris exclusion (FSC-A vs SSC-A) and doublet exclusion (FSC-A vs FSC-H), viable (FVD-) cells were gated for the murine PMN marker Ly6G.

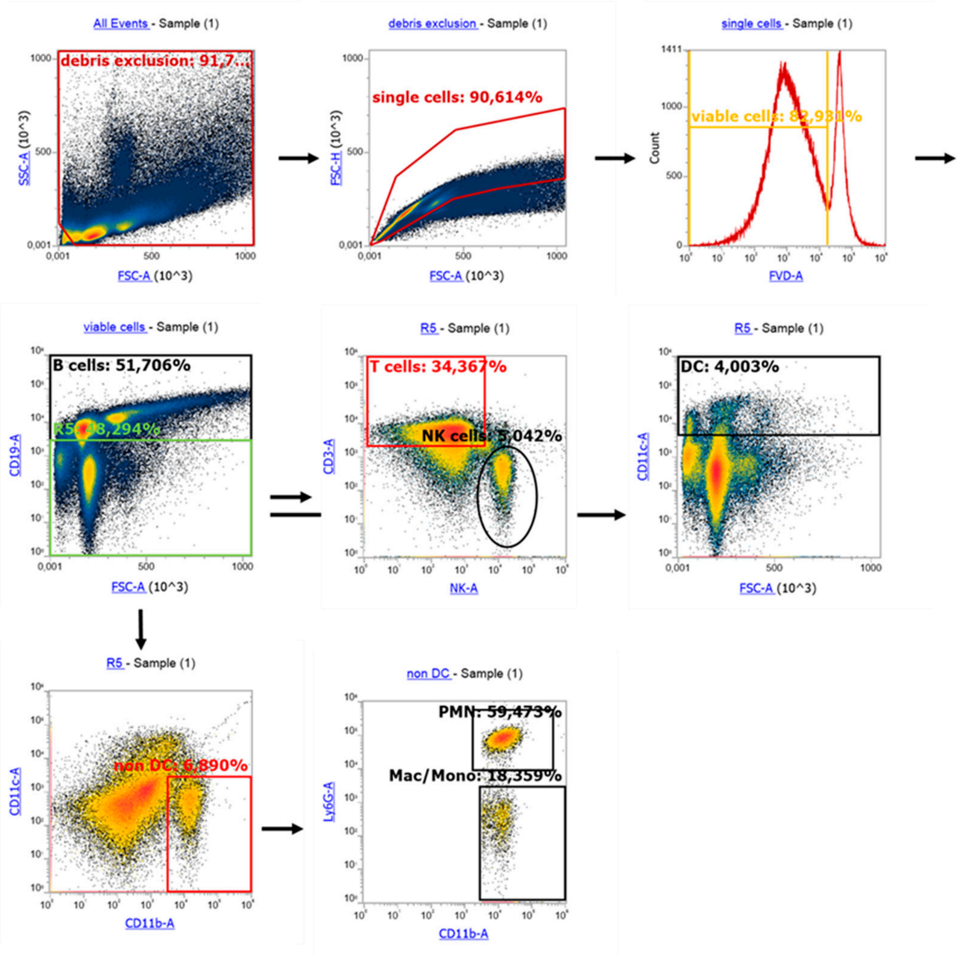

Figure S2. Flow cytometric gating strategy of splenocytes. Representative density plots and histograms of splenocyte populations are shown. After debris exclusion (FSC-A vs SSC-A) and doublet exclusion (FSC-A vs FSC-H), viable (FVD^-) cells were gated for B cells ($CD19^+$), T cells ($CD19^-NK1.1^-CD3^+$), NK cells ($CD19^-CD3^-NK1.1^+$), dendritic cells ($CD19^-CD11c^+$), PMN ($CD19^-CD11c^-CD11b^+Ly6G^+$) and macrophages/monocytes ($CD19^-CD11c^-CD11b^+Ly6G^-$).

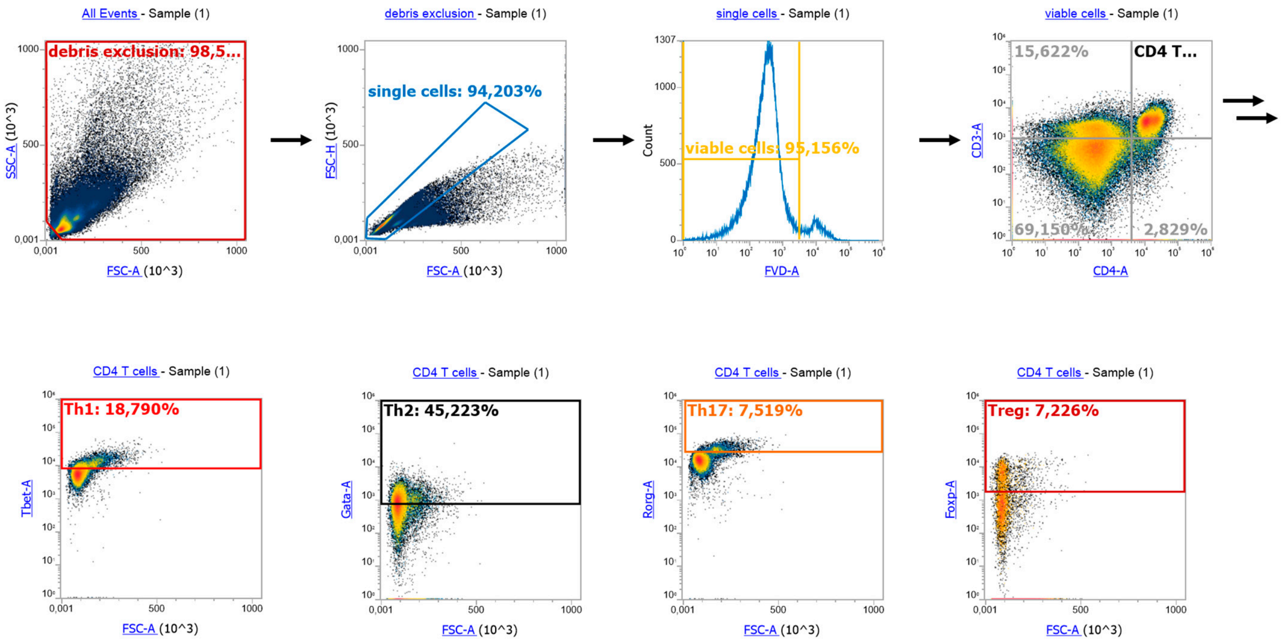

Figure S3. Flow cytometric gating strategy of $CD4^+$ T cell subsets. Representative density plots and histograms of splenocytes populations are shown. After debris exclusion (FSC-A vs SSC-A) and doublet exclusion (FSC-A vs FSC-H), viable (FVD^-) cells were gated for $CD4^+$ T cells ($CD3^+CD4^+$). These were further gated for Th1 (T-bet^+), Th2 (Gata-3^+), Th17 (Rorγt^+) and Treg (Foxp3+).

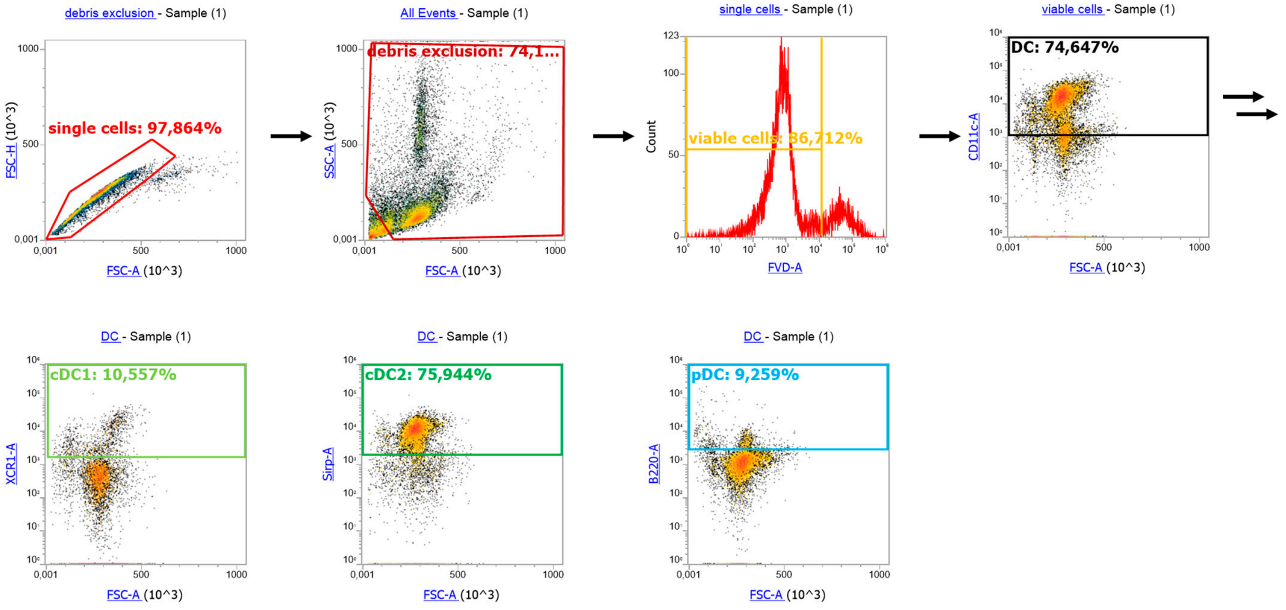

Figure S4. Flow cytometric gating strategy of primary dendritic cell subsets. Representative density plots and histograms of immunomagnetically isolated DC from spleens are shown. After debris exclusion (FSC-A vs SSC-A) and doublet exclusion (FSC-A vs FSC-H), viable (FVD-) cells were gated for the murine DC marker CD11c. DC subsets were identified by their expression of XCR1 (cDC1), Sirp1α (cDC2) or B220 (pDC).

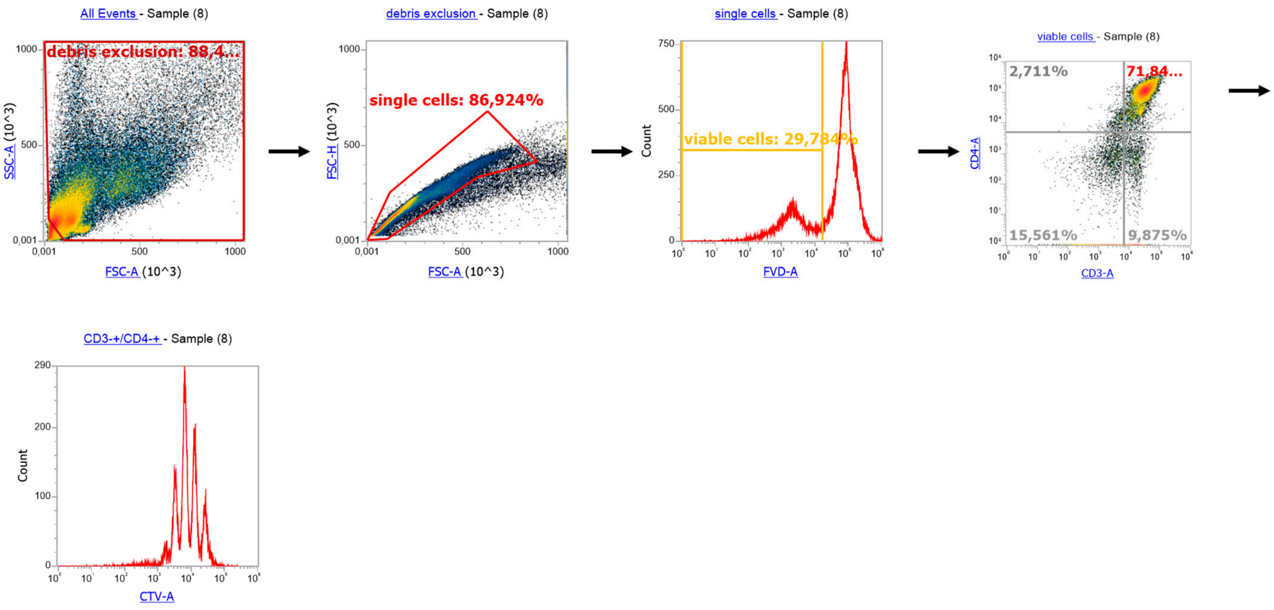

Figure S5. Flow cytometric gating strategy of CD4+ T cells after coculture with DCs. Representative density plots and histograms of cocultures comprising splenic DCs and Cell Trace Violet (CTV)-labeled $CD4^+$ T cells after coculture are shown. After debris exclusion (FSC-A vs SSC-A) and doublet exclusion (FSC-A vs FSC-H), viable (FVD^-) cells were gated for $CD3^+CD4^+$ and MFI of CTV was quantified.
